# Exposure to combustion generated environmentally persistent free radicals enhances severity of influenza virus infection

**DOI:** 10.1186/s12989-014-0057-1

**Published:** 2014-10-30

**Authors:** Greg I Lee, Jordy Saravia, Dahui You, Bishwas Shrestha, Sridhar Jaligama, Valerie Y Hebert, Tammy R Dugas, Stephania A Cormier

**Affiliations:** Department of Pharmacology and Experimental Therapeutics, Louisiana State University Health Sciences Center, New Orleans, Louisiana 70112 USA; Department of Pediatrics, University of Tennessee Health Sciences Center, Memphis, Tennessee 38103 USA; Children’s Foundation Research Institute, Le Bonheur Children’s Hospital, Memphis, Tennessee USA; Department of Pharmacology, Toxicology and Neuroscience, Louisiana State University Health Sciences Center, Shreveport, Louisiana 71103 USA

**Keywords:** Particulate matter, EPFR, Influenza, Infant, Neonate, Air pollution, DCB230, Oxidative stress

## Abstract

**Background:**

Exposures to elevated levels of particulate matter (PM) enhance severity of influenza virus infection in infants. The biological mechanism responsible for this phenomenon is unknown. The recent identification of environmentally persistent free radicals (EPFRs) associated with PM from a variety of combustion sources suggests its role in the enhancement of influenza disease severity.

**Methods:**

Neonatal mice (< seven days of age) were exposed to DCB230 (combustion derived PM with a chemisorbed EPFR), DCB50 (non-EPFR PM sample), or air for 30 minutes/day for seven consecutive days. Four days post-exposure, neonates were infected with influenza intranasally at 1.25 TCID_50_/neonate. Neonates were assessed for morbidity (% weight gain, peak pulmonary viral load, and viral clearance) and percent survival. Lungs were isolated and assessed for oxidative stress (8-isoprostanes and glutathione levels), adaptive immune response to influenza, and regulatory T cells (Tregs). The role of the EPFR was also assessed by use of transgenic mice expressing human superoxide dismutase 2.

**Results:**

Neonates exposed to EPFRs had significantly enhanced morbidity and decreased survival following influenza infection. Increased oxidative stress was also observed in EPFR exposed neonates. This correlated with increased pulmonary Tregs and dampened protective T cell responses to influenza infection. Reduction of EPFR-induced oxidative stress attenuated these effects.

**Conclusions:**

Neonatal exposure to EPFR containing PM resulted in pulmonary oxidative stress and enhanced influenza disease severity. EPFR-induced oxidative stress resulted in increased presence of Tregs in the lungs and subsequent suppression of adaptive immune response to influenza.

**Electronic supplementary material:**

The online version of this article (doi:10.1186/s12989-014-0057-1) contains supplementary material, which is available to authorized users.

## Background

The associations between PM pollution exposure and adverse health outcomes are well documented. These adverse health effects include respiratory diseases such as chronic obstructive pulmonary disease and asthma [1-5]. Interestingly, associations between PM exposure and increased susceptibility to infectious respiratory diseases are also observed [6-8]. Due to the heterogeneous nature of PM pollution, there is ongoing interest in identifying the particularly hazardous components. Recent studies by our collaborators identify the presence of “environmentally persistent free radicals” (EPFRs) in PM samples collected from various cities in the United States [9]. These newly identified EPFRs are a prime candidate as a causative agent for adverse outcomes associated with PM exposures, since free radicals are known to cause negative biological health effects.

Although PM exposure affects people of all ages, its effects on young children and infants are particularly detrimental [10,11]. Similarly, respiratory viral infections, such as seasonal influenza, also maintain an age bias where the very young and elderly are particularly at risk of more severe disease [12]. Infants are a unique and particularly susceptible population to both viral respiratory illnesses and PM pollution due to their developing pulmonary and immunological systems; and their faster breathing rates compared to adults.

There is a paucity of studies that delve into the biological mechanisms responsible for PM exposure-enhanced respiratory viral illness. Our prior studies demonstrate that exposure of neonatal mice (i.e., mouse age when pulmonary and immune system mimics a human infant [13,14]) to combustion derived PM induces both airway epithelial damage and barrier dysfunction and results in a temporary immunosuppressive pulmonary microenvironment [15,16]. The epithelial damage and/or the suppressed pulmonary immune microenvironment could be responsible for epidemiological data demonstrating enhanced morbidity following respiratory viral infection following exposure to elevated levels of PM pollution.

We hypothesized that EPFRs in the combustion derived PM are responsible for enhanced influenza morbidity and decreased survival observed in infants. We are studying the EPFR of dichlorobenzene that is formed by reaction with Cu(II)O containing fly-ash at 230°C (referred to as DCB230) [17,18]. To address our hypothesis, we exposed neonatal mice to EPFR containing PM (DCB230), non-EPFR containing PM (DCB50), or DCB230 in the presence of elevated antioxidants and subsequently infected them with influenza.

## Results

### Acute inhalation exposure to EPFRs induces pulmonary oxidative stress in neonates

**Figure 1:**
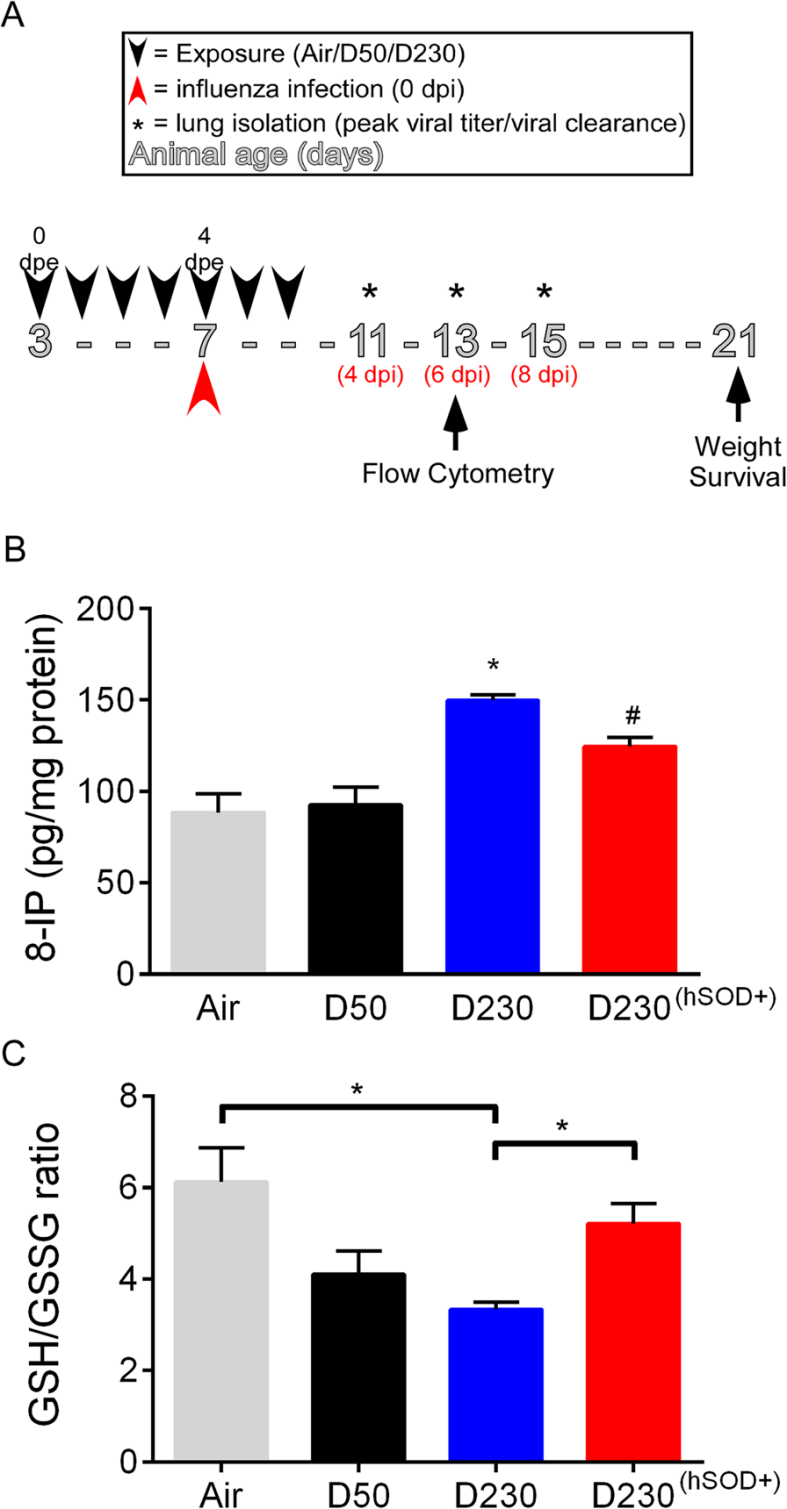
**Effect of EPFR exposure on oxidative stress burden in neonates.** **A)** Neonatal exposure and influenza infection model. Three day old mice were exposed to 200 μg/m^3^ of DCB230, DCB50, or air for seven consecutive days (0-7 dpe) for 30 min/day (black arrow head) and infected with influenza at 1.25 TCID50/neonate on the fourth dpe (red arrow head). Non-infected lungs were isolated at four dpe for analysis of oxidative stress and regulatory T cells (Tregs). Infected lungs were isolated for pulmonary viral load, flow cytometry, and pulmonary viral clearance on four, six, and eight days post-infection (dpi; asterisks). Pulmonary oxidative stress, determined by levels of **(B)** 8-isoprostance (IP) and **(C)** GSH/GSSG ratio in lungs of wild-type C57BL/6 after five days of exposure to air, DCB50 (D50), or DCB230 (D230) and in lungs of hSOD2 transgenic neonates exposed to DCB230 (D230^(hSOD+)^). Data plotted as mean ± standard error of mean (SEM). *p <0.05 D230 vs Air, D50, and D230^(hSOD+)^; # *p* <0.05 D230^(hSOD+)^ vs Air, D50, and D230; Brackets indicate *p* <0.05 D230 vs Air and D230^(hSOD+)^; one-way analysis of variance (ANOVA) with Tukey’s multiple comparisons test. N =4-10/group.

In a prior study, we observed oxidative stress in the lungs after seven days of exposure to DCB230 in neonatal rats [18] and bronchial epithelial cells [16]. To determine if oxidative stress was present earlier (within five days of exposure), three day old mice were exposed to air (Air), DCB50 (D50), or DCB230 (D230) (Figure 1A). After five days of exposure, we isolated the lungs and analyzed the lung homogenates for indicators of oxidative stress including 8-isoprostanes (8-IP) and glutathione/oxidized glutathione ratio (GSH/GSSG). Neonatal mice exposed to DCB230 had significantly higher levels of 8-IP compared to all other groups (Figure 1B). In addition, DCB230 exposed neonates had a significantly lower GSH/GSSG ratio compared to Air (Figure 1C). The GSH/GSSG ratio was not different between DCB230 and DCB50 exposed neonates. To help clarify the role of the EPFR in the induction of pulmonary oxidative stress, we also exposed neonatal mice that expressed human mitochondrial superoxide dismutase (hSOD^+^) to D230 (D230^(hSOD+)^). The additional antioxidant capacity was sufficient to significantly reduce the amount of 8-IP produced in the lungs during acute exposure to EPFRs and increase the ratio of GSH/GSSG. To ensure there were no inherent sub-strain differences between transgenic neonates and wild-type (WT) neonates, we compared oxidative stress responses in WT and hSOD2^-^ neonates exposed to DCB230. No differences in normalized levels of 8-IP between WT (D230/Air: 1.65 ± 0.054) vs. hSOD^-^ (D230^(hSOD-)^/Air^(hSOD-)^: 1.61 ± 0.18; Additional file 1: Figure S1A) or normalized GSH/GSSG ratios (D230/Air: 0.54 ± 0.03; D230^(hSOD-)^/Air^(hSOD-)^: 0.55 ± 0.07; Additional file 1: Figure S1B) was identified.

### Oxidative stress is critical in the enhancement of influenza morbidity observed following exposure to EPFRs in neonates

**Figure 2:**
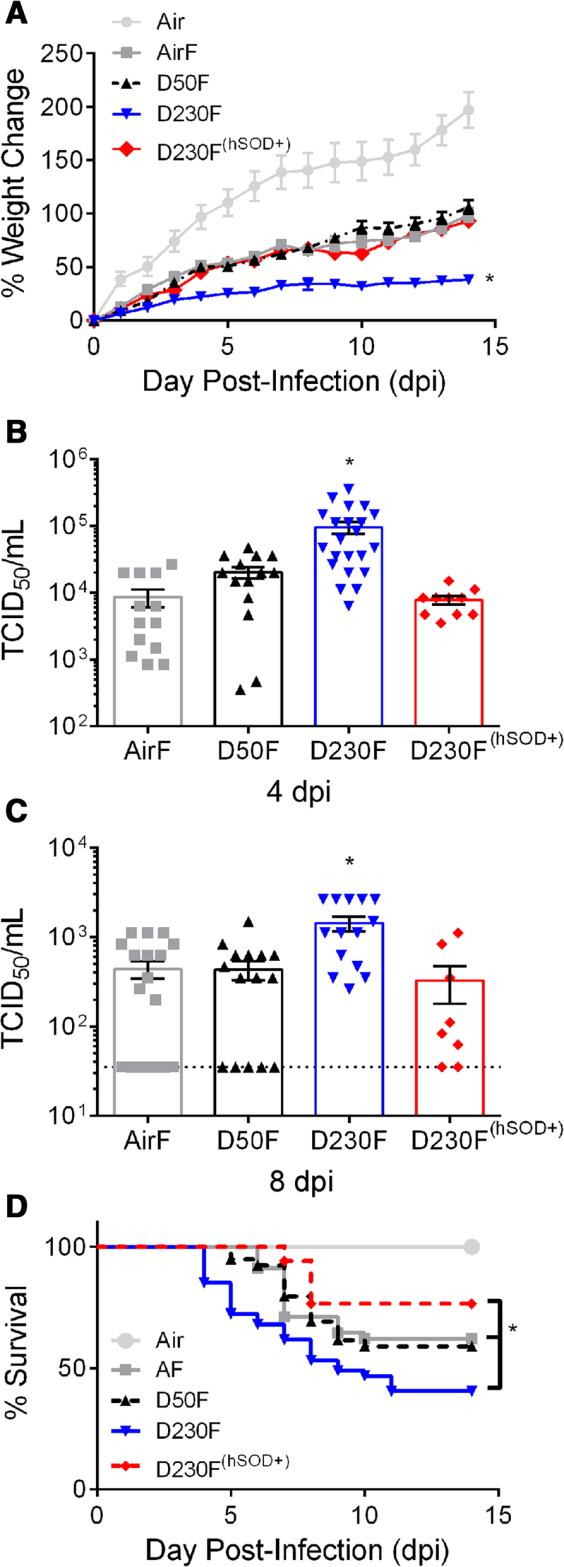
**Effect of EPFR exposure on influenza morbidity and viral load in neonates.** Neonatal mice were exposed to air, DCB50, or DCB230 and infected i.n. with influenza (AirF, D50F, D230F, D230F^(hSOD+)^) at 1.25 TCID_50_/neonate or sham infected with DPBS (Air). **(A)** Average percent change in weight plotted against dpi. N =16-35/group. **(B)** Peak pulmonary viral load assessed at four dpi. N =10-22/group. **(C)** Pulmonary viral clearance assessed at eight dpi. Dotted line indicates limit of detection (35 TCID_50_/mL) of assay. **(D)** Effect of EPFR exposure on influenza infection associated drop in survival in neonates. Survival was recorded daily until 14 dpi. N =8-18/group. Data plotted as mean ± SEM. *p <0.05 D230F vs Air, AirF, D50F, and D230F^(hSOD+)^. **(A)** Multiple t tests with Holm-Sidak correction for multiple comparisons. **(B and C)** One-way ANOVA with Tukey’s multiple comparisons test. **(D)** Gehan-Breslow Wilcoxon test with Bonferroni correction for multiple comparisons; *p <0.014 between D230F vs AirF and D230F^(hSOD+)^. N =17-47/group.

Epidemiological data demonstrate an increase in morbidity due to respiratory tract infections following high PM exposures [19-21]. Therefore, we assessed morbidity in the neonatal mice exposed to EPFRs. Mice were weighed daily to determine ability of influenza to induce weight loss and measured viral loads at the peak of viral replication and again four days later to assess the ability to control viral replication and to clear virus. Neonates were exposed to air or PM for seven consecutive days and sham (Air) or influenza (AirF, D50F, D230F) infected four days post-exposure (dpe; Figure 1A). Following infection, neonates exposed to DCB230 (D230F: 38.15 ± 4.57%; (Figure 2A) gained significantly less weight compared to all other groups (Air: 197.09 ± 16.74%; AirF: 98.91 ± 6.50%; D50F: 106.23 ± 6.54%). Higher pulmonary viral loads were also observed in DCB230 exposed neonates (D230F: 95007 ± 19657 TCID_50_/mL; Figure 2B) at four days post infection (dpi), which represents the peak viral load, compared to all other groups (AirF: 8626 ± 2573 TCID_50_/mL; D50F: 20198 ± 3829 TCID_50_/mL). Furthermore, DCB230 exposed neonates continued to present with significantly higher pulmonary viral loads at eight dpi (D230F: 1432 ± 265.5 TCID_50_/mL; Figure 2C) compared to all other groups (AirF: 440.7 ± 99.16 TCID_50_/mL; D50F: 434.1 ± 102.8 TCID_50_/mL). Of note, the exacerbation in morbidity (decreased weight gain and increased viral load at four and eight dpi) due to EPFR exposure was reversed in hSOD^+^ neonates (D230F^(hSOD+)^: 93.2 ± 6.94%; 7683 ± 1099 TCID_50_/mL; 327.8 ± 147.5 TCID_50_/mL). To ensure that these results were not due to sub-strain differences between WT and transgenic neonates, we also exposed WT and hSOD^-^ neonates to air and DCB230 with subsequent intranasal (i.n.) influenza infection. There were no observed differences in pulmonary viral load at four dpi between WT (Additional file 2: Figure S2A) and hSOD^-^ neonates exposed to DCB230 (AirF^(hSOD-)^: 7368 ± 3156 TCID_50_/mL; D230F^(hSOD-)^: 146343 ± 47621 TCID_50_/mL) as well as at eight dpi (AirF^(hSOD-)^: 266.3 ± 148.8 TCID_50_/mL; D230F^(hSOD-)^: 1512 ± 415.1 TCID_50_/mL; Additional file 2: Figure S2B). In fact, DCB230 exposed hSOD^-^ neonates were similar to their WT counterparts. Furthermore, there was no difference in pulmonary viral loads at four dpi between WT, hSOD^+^, or hSOD^-^ mice (AirF: 482.3 ± 62.34 TCID_50_/mL; AirF^(hSOD+)^: 1241 ± 496.8 TCID_50_/mL; AirF^(hSOD+)^: 431.7 ± 38.11 TCID_50_/mL; Additional file 3: Figure S3).

### EPFR exposure decreases survival following influenza infection

To assess the effects of EPFR exposure on mortality following influenza infection in neonates, we recorded daily survival data to 14 dpi. We observed a significant drop in percent survival in neonates exposed to D230F compared to AirF (40.43% vs. 62%; respectively; *p* <0.014) (Figure 2D). D50F exposed neonates had improved survival compared to D230F and was not statistically different from AirF. Again, the enhanced antioxidant capacity of hSOD^+^ neonates was able to significantly improve survival of mice exposed to DCB230 and infected with influenza (76.47% vs. 40.43%; respectively; *p* <0.014). Interestingly, D230F neonates consistently exhibited earlier mortality (four dpi) compared to all other groups.

### Protective immune responses against influenza are diminished following EPFR exposure

**Figure 3:**
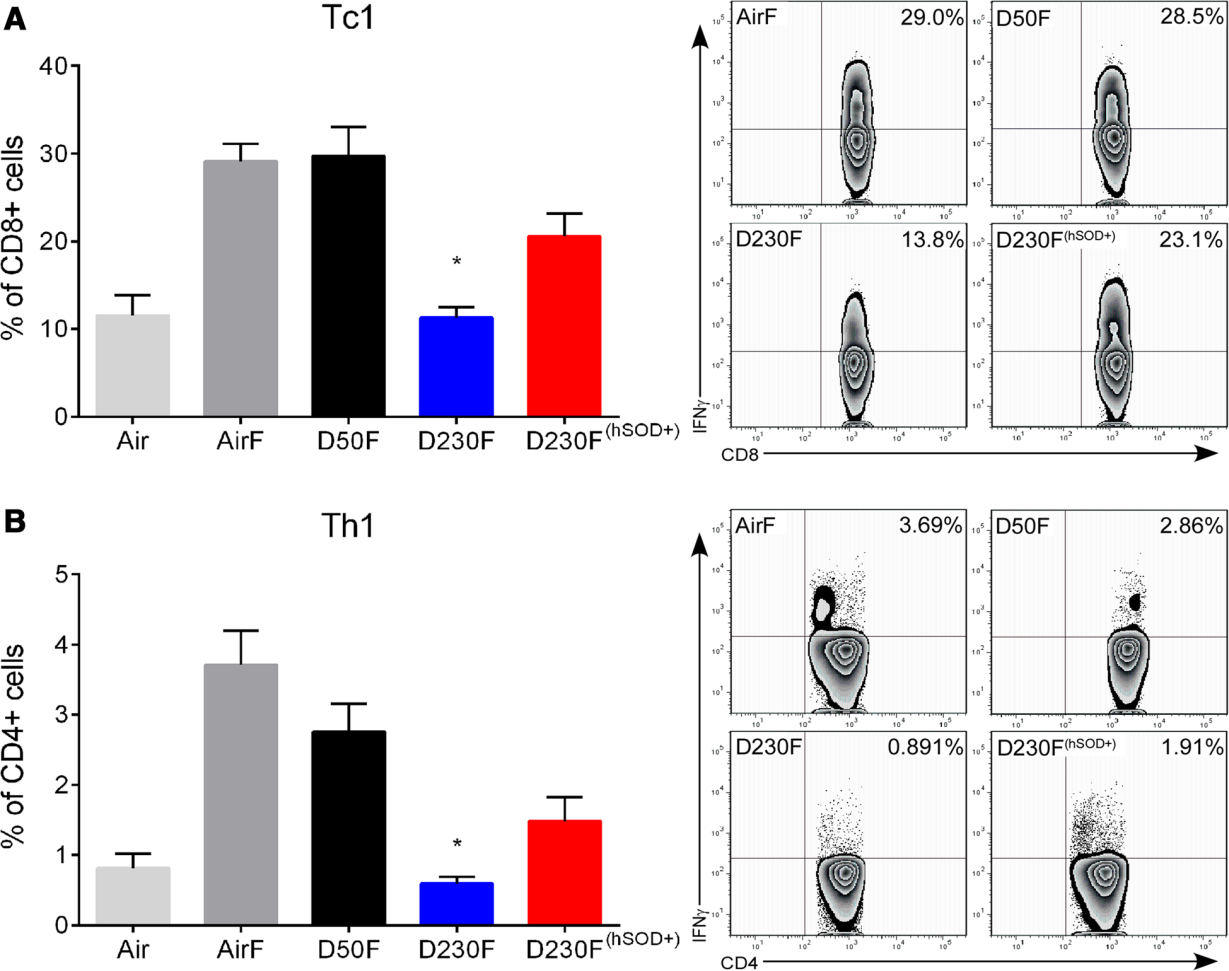
**Effect of EPFR exposure on adaptive immune response (Tc1 and Th1) to influenza infection in neonates.** Neonatal mice were exposed to air, DCB50, or DCB230 and infected i.n. with influenza (AirF, D50F, D230F, D230F^(hSOD+)^) or sham infected with DPBS (Air). Lung effector T cell profiles were determined by flow cytometry at six dpi. **(A)** Percentage of CD8+ cells expressing IFNγ (Tc1) with representative flow contour plots demonstrating outliers. N = 3-12/group. **(B)** Percentage of CD4+ cells expressing IFNγ (Th1) with representative flow contour plots demonstrating outliers. N = 3-12/group. Data plotted as mean ± SEM. *p <0.05 D230F vs AirF, D50F, and D230F^(hSOD+)^; multiple t tests with Holm-Sidak correction for multiple comparisons.

To determine the effects of EPFR exposure on the adaptive immune response to influenza, lungs were isolated at six dpi and T cell profiles were assessed. T cells expressing IFNγ are critical to control and clear influenza. The percent of CD8+ T cells expressing IFNγ (Tc1) were significantly reduced in D230F neonates (Figure 3A) compared to AirF or D50F. Furthermore, the percent of CD4+ T cells expressing IFNγ (Th1) were significantly lower in D230F neonates (Figure 3B) compared to AirF and D50F. The increased antioxidant capacity afforded to D230F^(hSOD+)^ neonates resulted in significant increases in the percentage of both the Tc1 and Th1 cells compared to D230F. To further confirm whether percent Tc1 and Th1 values show similar trends in terms of total number of lung Tc1 and Th1 cells, the percent data was transformed to and analyzed in terms of total lung Tc1 and Th1 cells. Similar to percent data, the total number of both Tc1 and Th1 cells were significantly reduced in D230F neonates compared to AF or D50F and increased antioxidant capacity in D230F^(hSOD+)^ neonates resulted in a significant increase in total lung Tc1 and Th1 cells compared to D230F (Additional file 4: Figure S4).

### Oxidative stress following exposure to EPFRs induces an increase in regulatory T cells in the lungs

**Figure 4:**
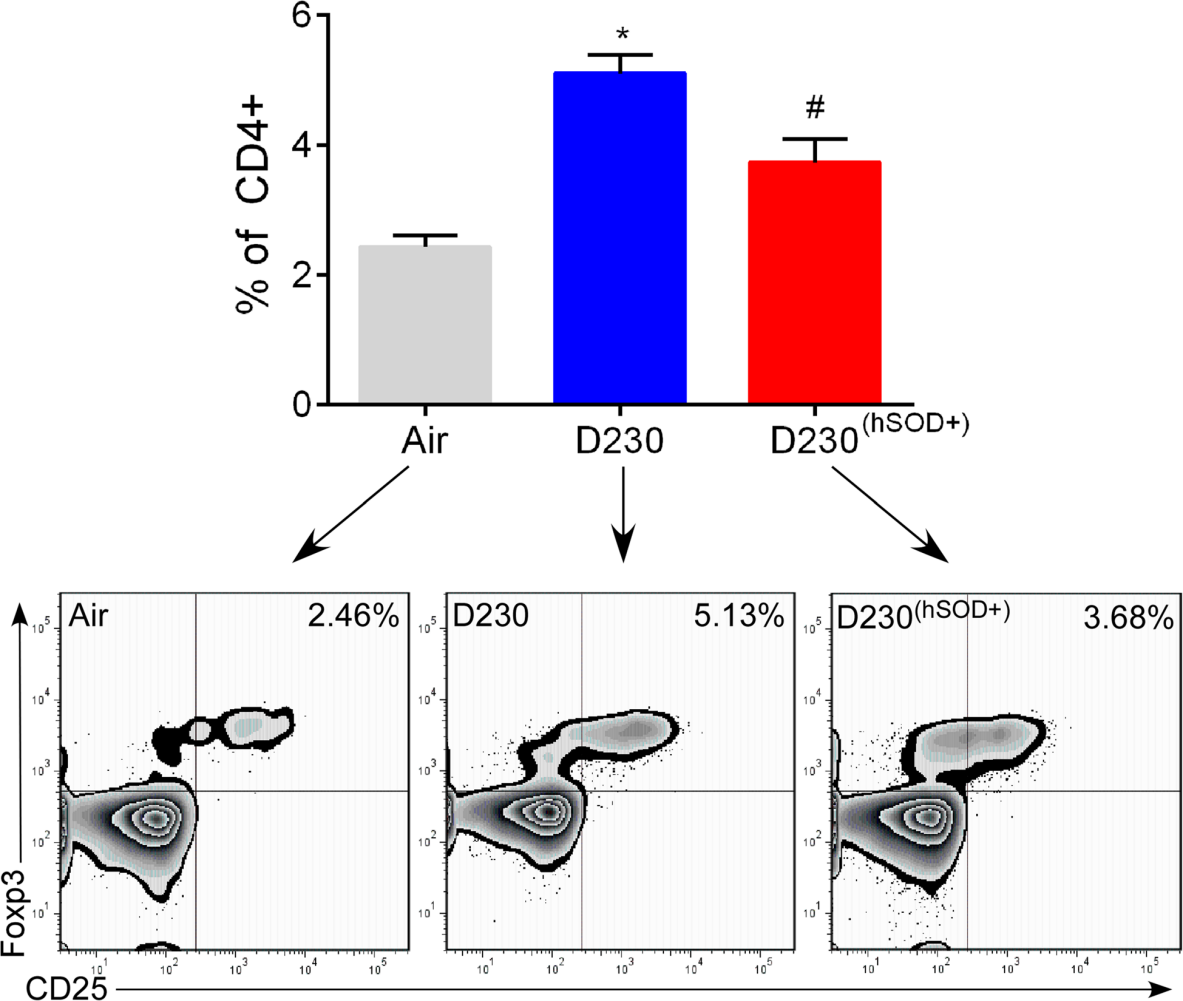
**Effect of EPFR exposure on pulmonary regulatory T cells (Tregs).** Neonatal mice were exposed to air or DCB230 (Air, D230, D230^(hSOD+)^) for five consecutive days and lungs were isolated four dpe (at the time infection would have occurred). Pulmonary Tregs were identified and quantified by flow cytometry. N =6-8/group. Data plotted as mean ± SEM with representative flow contour plots with outliers. *p <0.05 D230 vs Air and D230^(hSOD+)^, # *p* <0.05 D230^(hSOD+)^ vs Air and D230; one-way ANOVA with Tukey’s multiple comparisons test.

Our previous data demonstrated that acute exposure to combustion derived PM induced a population of immunosuppressive T cells - Tregs [15]. However, from those studies it was unclear if this was due to the presence of EPFRs in the PM samples or due to epithelial injury. To determine if exposure to EPFRs induced a Treg response, we isolated lung cells at four dpe (i.e., the time of maximal epithelial injury and at the time influenza infection would have occurred in our model; Figure 1A) and quantified Tregs using flow cytometry. We observed significant increases in the percentage of Tregs in the lungs of neonates exposed to DCB230 compared to Air (Figure 4). The expression of hSOD2 resulted in a significant decrease in the percentage of pulmonary Tregs compared to D230; however, hSOD2 expression was not able to return Tregs numbers to homeostatic baseline levels.

## Discussion

The association between PM exposure and enhanced respiratory viral disease severity in children is well documented. Studies that have investigated this phenomenon in adult animals have given important insights to this association [22], but it is also important to understand these responses in vulnerable subpopulations such as infants. Our studies, along with others, show that response (immunological or cellular) to agents (infectious and non-infectious) is markedly different between adults and neonates [14,15,23-28]. Our lab has already identified that pre-natal EPFR exposure increases the risk of asthma development in offspring [29]. Since infants are still undergoing dramatic pulmonary and immunological development, insults during this critical time period may incur additional and long term health consequences. Furthermore, the discovery of EPFRs present in real world PM from almost all combustion sources suggests its role in the adverse health effects associated with PM exposure [30]. This manuscript presents a novel model to study the effects of PM exposure and influenza infection in neonates, as well as, explores the effects of airborne EPFR exposure in neonates on influenza severity. Our neonatal model recapitulated what has been observed in epidemiological studies – enhanced severity of respiratory tract viral infection following exposure to elevated levels of PM [19-21,31]. In addition, our data demonstrated that exposure to PM containing EPFRs induced oxidative stress in neonates. This oxidative stress could be attenuated by increasing the antioxidant capacity of the host. Furthermore, our data revealed specifically that EPFR exposure, and not just PM exposure (DCB50), exacerbated morbidity due to influenza as evidenced by reductions in weight gain and higher pulmonary viral loads at both four and eight dpi (peak viral titer and viral clearance, respectively). More strikingly, EPFR exposure induced premature and decreased survival following influenza infection. Increased antioxidant capacity of the host was able to attenuate EPFR-enhanced morbidity and enhance survival following influenza infection.

Since the adaptive immune response is paramount in the host defense against pathogens, we focused our studies on the subset of CD8+ and CD4+ T cells instrumental in controlling and clearing intracellular pathogens such as influenza [32,33]. We discovered that both the subset of CD8+ and CD4+ T cells that release IFNγ (Tc1 and Th1, respectively) were significantly decreased following EPFR exposure in neonates. Due to the observed immunosuppressed phenotype in EPFR exposed and influenza infected neonates, we investigated the role of Tregs in EPFR exposed non-infected neonates because of their known role in immunosuppression. Our previously published data showed increased Treg numbers that coincided with increased epithelial injury even in the absence of influenza infection indicating that Tregs are not increased with increased viral load but instead they are induced by epithelial injury [34]. To determine whether Tregs induced solely due to EPFR exposure are responsible for the immunosuppressive environment in the lungs, we analyzed Treg responses prior to influenza infection (four dpe), at the time of maximum lung injury. Our data revealed that EPFR exposure alone significantly increased the percentage of Tregs in the lungs of neonates. This increase in Tregs could potentially explain the dampened Tc1 and Th1 effector response in influenza infected neonates exposed to EPFRs. In fact, in our prior study, we observed a similar phenomenon – early exposure to EPFRs resulted in Treg expansion and suppression of T cell responses to allergen sensitization and challenge (i.e., mouse asthma model) [15]. Importantly, Treg expansion in this model, although transient, appeared to create a window of vulnerability to infection. Indeed, our data presented here support our prior findings and clearly demonstrate that neonatal mice exposed to EPFRs are more vulnerable to influenza infection and exhibit enhanced morbidity and decreased percentage survival following infection. Cumulatively, our data suggest that this window of vulnerability resulting from exposure to EPFR-containing PM will dampen the ability to mount protective adaptive immune responses to any pathogen and would ultimately lead to inability to clear the pathogen and more severe disease, as was observed here.

Our data also demonstrated that exposure to PM containing EPFRs induced both oxidative damage (as evidenced by increased levels of 8-IP) and redox imbalances (as evidenced by decreased GSH/GSSG ratios) in the lungs. Together these markers suggest that the lung was in a state of oxidative stress prior to infection. To determine the association between oxidative stress in the enhanced influenza severity following EPFR exposure, neonatal mice were exposed to non-EPFR containing PM (DCB50) and we included neonates with a higher antioxidant capacity throughout our studies (hSOD^+^). D50 and D230^(hSOD+)^ neonates exhibited lower pulmonary oxidative stress. Even more exciting, our data showed that PM exposure in the absence of an EPFR, or in the presence of an EPFR but within a host with enhanced antioxidant capacity, resulted in significantly less morbidity and enhanced survival in neonatal mice following infection with influenza. The improvement in morbidity and survival was associated with an improvement in the adaptive immune response to influenza (Tc1 and Th1) and a reduction in immune regulating T cells (Tregs). Taken together, our data identified pulmonary oxidative stress as a key component in the pathogenesis of PM enhanced influenza disease severity.

A related study by another laboratory looking at influenza viral loads in adult mice after exposure to diesel exhaust observed similar increases in viral load [22]. Although both studies identified increased severity of influenza mediated disease, only our study demonstrated a significant benefit associated with elevated antioxidant capacity (i.e., decreased pulmonary viral load and improved survival). In addition, we were able to show that oxidative stress induced by EPFR exposure resulted in immunosuppression, which could be alleviated by increasing the antioxidant capacity during the exposure resulting in enhanced protective immune responses. These incongruences could be explained by a myriad of differences between our studies including age (adult vs. neonate), strain of the mice (BALB/c vs. C57Bl/6), timing of the exposure to diesel exhaust or EPFR containing PM (post-infection vs. before and during infection), and the type, dose, and route of antioxidant utilized (N-acetylcysteine vs. SOD2).

Other studies have attempted to explain how exposure to PM exacerbates respiratory viral infections in neonates as a model to understand similar findings in human infants. Some have speculated that PM facilitates the spread of influenza by acting as condensation nuclei for viral droplets [35]. Other studies report enhanced viral attachment due to exposure to diesel exhaust [36], another source of EPFRs. The role of oxidative stress has also been studied and investigators report that oxidative stress inhibits GSH [37] which can lead to enhanced virus replication [38]. Oxidative stress can also inhibit mucus antiproteases [39] that regulate the trypsin like serine proteases which cleaves influenza hemagluttinin, effectively enhancing its ability to spread and more readily infect epithelial cells [40,41]. Our study proposes an additional mechanism for this phenomenon. First, our data suggest that exposure to PM containing EPFRs, which is found in most combustion derived PM, is responsible for the exacerbation of influenza in neonates. In addition, EPFR exposure resulted in enhanced pulmonary oxidative stress that led to increased pulmonary Tregs prior to infection. Our previous data suggested that the increased Tregs in the lungs results in an immunosuppressive environment that subsides within a week if exposure to EPFRs is discontinued [15]. We believe that the persistence of this immunosuppressive environment during and throughout the infection dampens the development of a protective adaptive immune response to influenza infection. Ultimately this is responsible for the increased morbidity and decreased survival observed. The effects of EPFR exposure were subsequently attenuated when antioxidant capacity was increased.

## Conclusions

We demonstrate that EPFRs associated with combustion derived PM are important in enhancing severity and mortality following respiratory tract viral infections. Our data demonstrate the benefit of antioxidant supplementation in reversing the enhanced disease due to influenza infection in the context of EPFR-containing PM exposure. The antioxidant data further suggest that it may be clinically beneficial to identify at-risk infants (i.e., those who reside in high traffic areas, reside in homes with smokers, live in homes where biomass fuels are used, etc.), so that supplementation with antioxidants prior to influenza season may be employed to reduce disease burden. It is important to note that the results from this study only suggest the benefits of prophylactic usage of antioxidants prior to/concurrent with exposure to EPFRs. It is worthwhile to study the effectiveness of antioxidants as a therapeutic agent, post-exposure and potentially post-infection. Finally, it would be interesting to investigate the role of EPFR exposure and secondary or concurrent bacterial/viral infection in neonates as mortality incidences are astronomically high in these patients. In summary, this study illustrates a unique neonatal model to further investigate the mechanism by which air pollution modulates respiratory viral illnesses and disease. This model can easily be extrapolated to other non-viral or non-pathogenic insults that infants commonly encounter.

## Methods

### Combustion derived PM

PM with a mean aerodynamic diameter of 0.2 μm was generated and characterized by our colleague (Dr. Slawo Lomnicki, LSU) as previously described [17,18]. Briefly, combustion derived PM containing a chemisorbed EPFR (DCB230) or lacking an EPFR (DCB50) was suspended in 25 mL of irrigation saline containing 0.02% Tween-80 resulting in a final concentration of 0.2 mg/mL. The particles were monodispersed via probe sonication immediately prior to use.

### Animals

C57BL/6NHsd breeders were purchased from Harlan (Indianapolis, IN). Human SOD2 transgenic mice were generously provided by Nils-Goran Larsson of the Max Planck Institute for Biology of Ageing (Cologne, Germany). All animals were housed in ventilated cages in a specific pathogen free environment and time mated to maximize birth cohorts. Pups expressing the hSOD2 transgene (hSOD^+^) were generated by crossing heterozygous hSOD2/+ male mice to wild-type C57BL/6 (WT) females. The presence of the transgene was confirmed by PCR. Littermates not expressing hSOD2 (hSOD^-^) were used as controls. Separate cohorts of pups were used for independent experimental end points (oxidative stress or flow cytometry or viral load). Neonatal mice were not identified for sex; and therefore, mice of both sexes were used for all the experiments. All animal protocols were prepared in accordance with the Guide for the Care and Use of Laboratory Animals and approved by the LSUHSC and UTHSC Institutional Animal Care and Use Committee.

### Neonatal exposures

Neonatal mice, defined as less than seven days of age, were exposed to air, DCB50, or DCB230. To mimic a human infant exposure, we relied on modeling data (MPPD v2.0) to produce an equivalent particle deposition per alveolus to that of an infant human being exposed over a 24 h time period to an average of 35 ug/m^3^ of PM2.5. Thus the whole body exposure dose was set to 200 μg/m^3^. The parameters used in the algorithm and how the air flow in the chamber were calculated and monitored were described previously [15]. A 3 day old mouse mimics a newborn human infant in regards to pulmonary development [13]. Hence, to mimic the exposure of a newborn human infant to particulate matter containing EPFRs, neonatal mice were exposed to EPFRs beginning on day 3 of age. Mice were placed in a chamber system in which particles were nebulized for 30 minutes/day for seven consecutive days reaching an average daily exposure concentration of 200 μg/m^3^. These exposure conditions were selected to mimic real-world exposure scenarios and were based on modeling calculations to achieve an equivalent alveolar deposition dose as in human infants as previously described [15].

### Neonatal influenza model

Neonatal pups were infected four dpe i.n. with 1.25 TCID_50_/neonate of mouse adapted human influenza A strain PR/8/34 H1N1 (Advanced Biotechnologies, MD) in 10 μL of Dulbecco’s phosphate-buffered saline (DPBS). Non-infected neonates were dosed with the same volume of DPBS. Weight gain and survival were assessed daily, immediately following infection, for the duration of the protocol (Figure 1).

### Markers of oxidative stress (8-isoprostanes, GSH/GSSG ratio)

Lungs were excised after five days of exposure (at the time infection would have occurred) and immediately flash frozen in liquid nitrogen for analysis for 8-IP and GSH/GSSG. Samples were saponified and purified as described previously [42] and 8-IP was quantified by ELISA (Cayman, MI) as per the manufacturer’s protocol. GSH/GSSG was detected using HPLC electrochemical detection as previously described [16].

### Pulmonary viral load

Lungs were excised and isolated at four dpi and eight dpi. Lungs were homogenized and viral titer was determined using the TCID_50_ method of Spearman and Kärber [43,44]. Briefly, Madin-Darby canine kidney cells were seeded at a density of 1.5 × 10^4^ cells/well on a 96 well flat bottom plate. Cells were subsequently inoculated with a 10-fold dilution series of homogenized lungs. Cells were incubated at 37°C with 5% CO_2_ for five days, observed daily for signs of cytopathic effect. Scoring for cytopathic effect was finalized on day five and TCID_50_ calculated.

### Flow cytometry and intracellular staining

Pups were sacrificed and blood was gently flushed from the lungs by retrograde perfusion with isotonic saline. A single lung cell suspension was prepared as previously described [15,25,45]. Briefly, perfused lungs were isolated, collected in chilled Hank’s balanced salt solution (HBSS), and mechanically dissociated using the Octodissociator (Miltenyi, Germany). Dissociated lungs were incubated and digested at 37°C with continuous shaking (200 rpm) for 30 minutes in HBSS supplemented with 1 mg/mL collagenase I (Invitrogen, NY), and 150 ng/mL DNase I (Sigma Aldrich, MO). Following incubation, lungs were dissociated a second time with the Octodissociator and remaining cell clumps dissociated by straining through a 40 μm cell strainer (BD Biosciences, CA). The resulting single cell lung suspension was further treated with RBC lysis buffer to remove residual red blood cells. Cells were stimulated for intracellular cytokine staining for five hours at 37°C in RPMI 1640 media containing 5% heat-inactivated fetal bovine serum, 5 ng/mL phorbol 12-myristate 13-acetate, and 500 ng/mL ionomycin (Sigma-Aldrich) in the presence of a protein transport inhibitor (GolgiPlug, BD Biosciences). For T cell staining, the following antibodies were used: eFluor450-CD3 (17A2; eBiosciences, CA), FITC-CD8 (53-6.7; eBiosciences), PerCP-CD4 (RM4-5; BioLegend, CA), and PE-IFNγ (XMG1.2; eBiosciences). For regulatory T cell staining, the following antibodies were used: eFluor450-CD3 (17A2; eBiosciences), PerCP-CD4 (RM4-5; BioLegend), PE-CD25 (PC61.5; eBiosciences), FITC-Foxp3 (FJK-16s; eBiosciences). Dead cells were excluded using a fixable live/dead marker eFluor 780 (eBiosciences). Stained cells were analyzed with a FACS Canto II (BD Biosciences). Flow data were analyzed and plotted using FlowJo Software v.7.6.5 (Tree Star, OR).

### Statistical analysis

All results are expressed as mean ± SEM and were analyzed using GraphPad Prism 6 (GraphPad Software Inc., Version 6.03, CA). One way analysis of variance (ANOVA) with Tukey’s multiple comparisons tests, multiple t tests with Holm-Sidak correction for multiple comparisons, and unpaired t test were used to determine differences between groups. Differences between survival curves were analyzed using Gehan-Breslow Wilcoxon test with Bonferroni correction for multiple comparisons. P values less than 0.05 were considered statistically significant, except where noted (survival curve analysis).

## Additional files

Additional file 1: Figure S1. EPFR exposure induces oxidative stress in hSOD2 negative (hSOD2^-^) littermate control neonates. WT or hSOD^-^ neonates were exposed to air or DCB230 (Air, D230, Air^(hSOD-)^, D230^(hSOD-)^). Pulmonary oxidative stress was determined by assessing levels of (A) 8-IP and (B) GSH/GSSG after five days of exposure. N =4-10/group. Data from mice exposed to DCB230 was normalized to respective Air controls, data plotted as mean ± SEM. *p* >0.05; Unpaired t test. Additional file 2: Figure S2. Effect of EPFR exposure on influenza morbidity in hSOD2^-^ littermate control neonates. Neonates were exposed to Air or DCB230 and infected i.n. with influenza (AirF, D230F) at 1.25 TCID_50_/neonate. (A) Pulmonary peak viral titer assessed at four dpi. N =5-22/group. (B) Pulmonary viral clearance assessed at eight dpi. N =6-18/group. Data plotted as mean ± SEM. *p* <0.05 (brackets); one-way analysis of variance (ANOVA) with Tukey’s multiple comparisons test. Additional file 3: Figure S3. Peak pulmonary viral load assessed in WT (AirF), hSOD2 transgenic (h + AirF), and transgenic littermates lacking hSOD2 (h-AirF) neonates at four days post-infection. Neonates were infected i.n. with influenza at 1.25 TCID_50_/neonate and peak pulmonary viral load was assessed at four dpi. N =6-7/group. Dotted line indicates limit of detection (35 TCID_50_/mL) of assay. Data plotted as mean ± SEM. *p* >0.05; Dunn’s multiple comparisons test. Additional file 4: Figure S4. Effect of EPFR exposure on pulmonary adaptive T cell responses (Tc1 and Th1) after infection to influenza in neonatal mice. Mice were exposed to air, DCB50, or DCB230 and infected i.n. with influenza (AirF, D50F, D230F, D230F^(hSOD+)^) or sham infected with DPBS (Air) at four dpe. Total lung effector T cell profiles were determined by flow cytometry at six dpi. (A) Total number of CD8+ cells expressing IFNγ (Tc1) with representative flow contour plots demonstrating outliers. N = 3-12/group. (B) Total number of CD4+ cells expressing IFNγ (Th1) with representative flow contour plots demonstrating outliers. N =3-12/group. Data plotted as mean ± SEM. **p* <0.05 D230F vs AirF, D50F, and D230F^(hSOD+)^; multiple t tests with Holm-Sidak correction for multiple comparisons.

## Abbreviations

EPFR: Environmentally persistent free radicals
DCB230: EPFR containing PM
SOD2: Superoxide dismutase 2, hSOD^+^, transgenic mouse expressing human SOD2
DCB50: PM lacking EPFR
8-IP: 8-isoprostane
GSH/GSSG: Glutathione/glutathione disulfide
hSOD^-^: Littermate not expressing human SOD2
dpi: Day post-infection
i.n.: Intranasal
Tc1: CD8+ T cell expressing IFNγ
Th1: CD4+ T cell expressing IFNγ
Tregs: Regulatory T cells
DPBS: Dulbecco’s phosphate-buffered saline
HBSS: Hank’s balanced salt solution
SEM: Standard error of means
ANOVA: Analysis of variance
