# Supplementary figures and images for: Exposure to combustion generated environmentally persistent free radicals enhances severity of influenza virus infection
Source: Part Fibre Toxicol. 2014 Oct 30;11:57. doi: 10.1186/s12989-014-0057-1 (PMC4222384; doi:10.1186/s12989-014-0057-1)

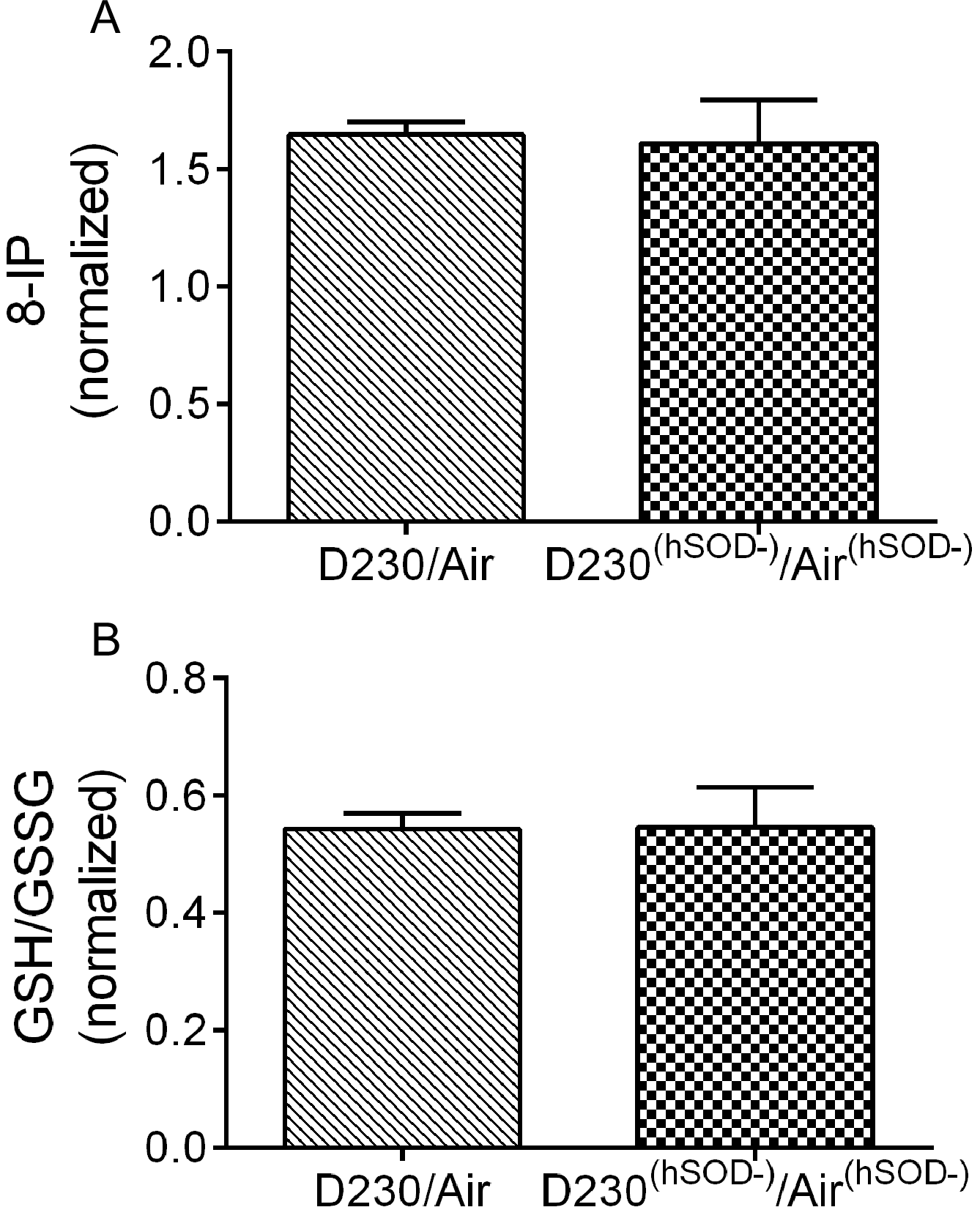

Supplement: Additional file 1: Figure S1. — EPFR exposure induces oxidative stress in hSOD2 negative (hSOD2-) littermate control neonates. WT or hSOD- neonates were exposed to air or DCB230 (Air, D230, Air(hSOD-), D230(hSOD-)). Pulmonary oxidative stress was determined by assessing levels of (A) 8-IP and (B) GSH/GSSG after five days of exposure. N =4-10/group. Data from mice exposed to DCB230 was normalized to respective Air controls, data plotted as mean ± SEM. p >0.05; Unpaired t test. [file 12989_2014_57_MOESM1_ESM.tiff]

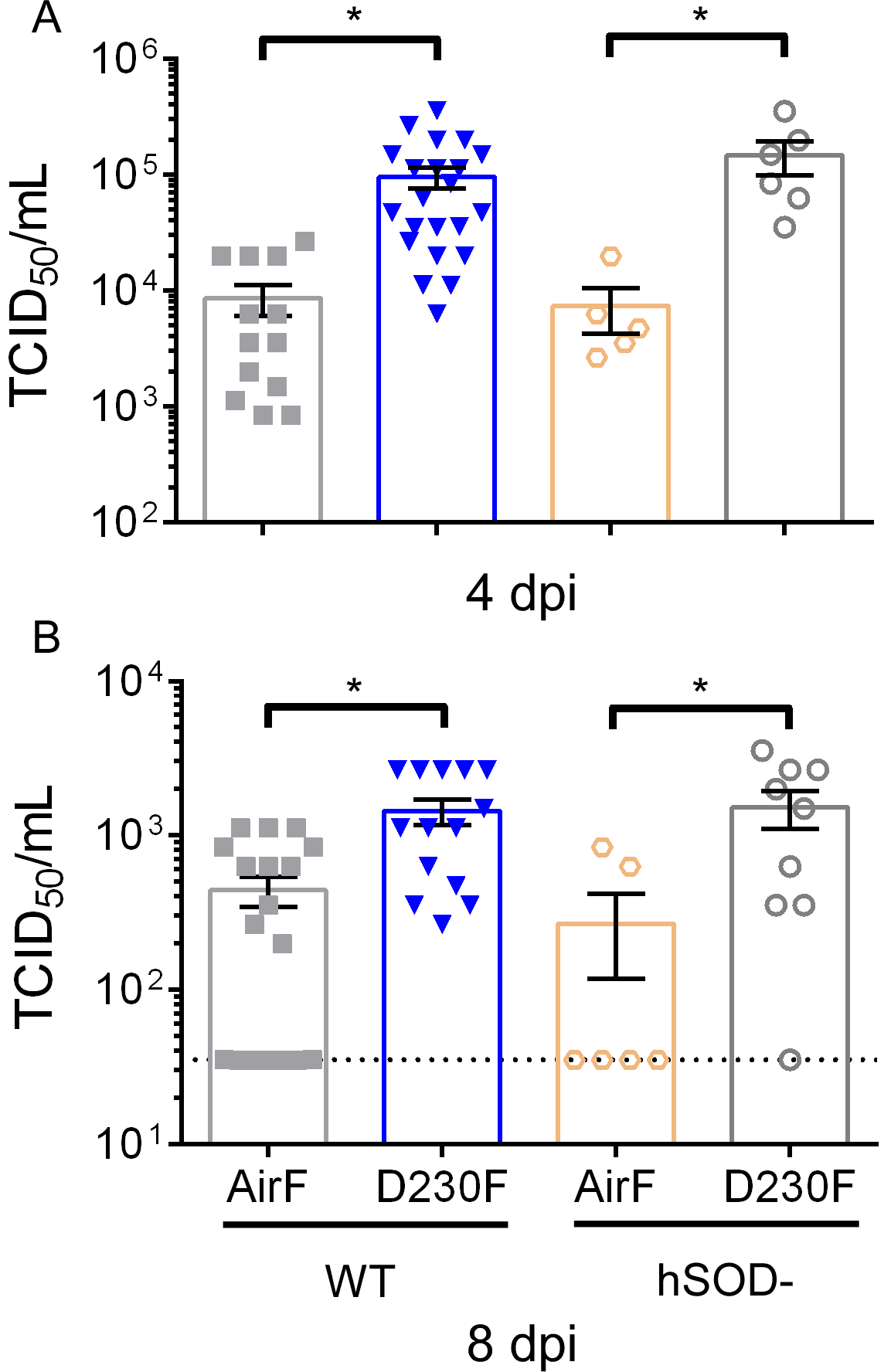

Supplement: Additional file 2: Figure S2. — Effect of EPFR exposure on influenza morbidity in hSOD2- littermate control neonates. Neonates were exposed to Air or DCB230 and infected i.n. with influenza (AirF, D230F) at 1.25 TCID50/neonate. (A) Pulmonary peak viral titer assessed at four dpi. N =5-22/group. (B) Pulmonary viral clearance assessed at eight dpi. N =6-18/group. Data plotted as mean ± SEM. p <0.05 (brackets); one-way analysis of variance (ANOVA) with Tukey’s multiple comparisons test. [file 12989_2014_57_MOESM2_ESM.tiff]

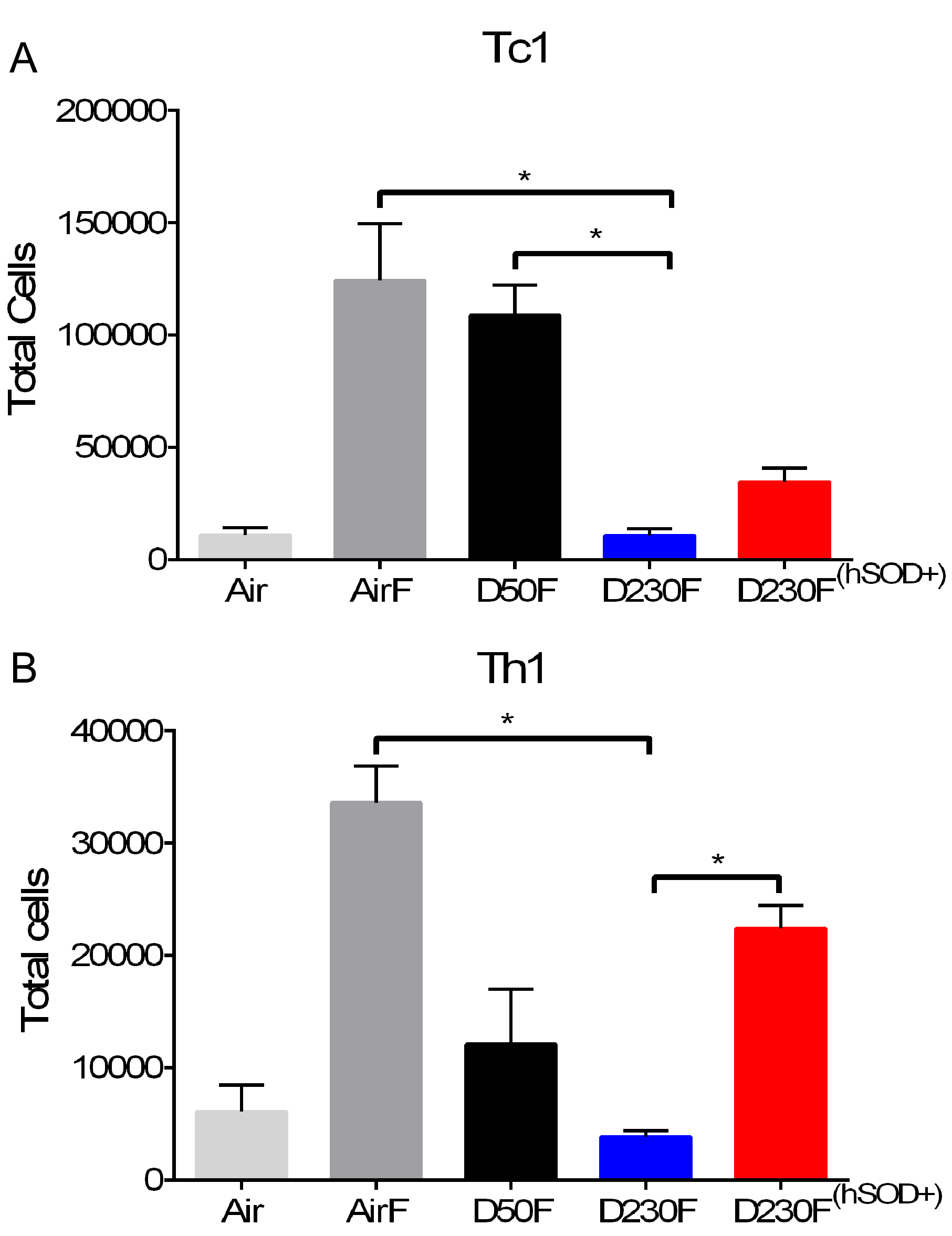

Supplement: Additional file 4: Figure S4. — Effect of EPFR exposure on pulmonary adaptive T cell responses (Tc1 and Th1) after infection to influenza in neonatal mice. Mice were exposed to air, DCB50, or DCB230 and infected i.n. with influenza (AirF, D50F, D230F, D230F(hSOD+)) or sham infected with DPBS (Air) at four dpe. Total lung effector T cell profiles were determined by flow cytometry at six dpi. (A) Total number of CD8+ cells expressing IFNγ (Tc1) with representative flow contour plots demonstrating outliers. N = 3-12/group. (B) Total number of CD4+ cells expressing IFNγ (Th1) with representative flow contour plots demonstrating outliers. N =3-12/group. Data plotted as mean ± SEM. *p <0.05 D230F vs AirF, D50F, and D230F(hSOD+); multiple t tests with Holm-Sidak correction for multiple comparisons. [file 12989_2014_57_MOESM4_ESM.tiff]
